# Contributions of bilateral white matter to chronic aphasia symptoms as assessed by diffusion tensor MRI

**DOI:** 10.1016/j.bandl.2015.09.001

**Published:** 2015-11

**Authors:** Sharon Geva, Marta M. Correia, Elizabeth A. Warburton

**Affiliations:** a Department of Clinical Neurosciences, University of Cambridge, United Kingdom; b Cognitive Neuroscience and Neuropsychiatry Section, UCL Institute of Child Health, United Kingdom; c MRC Cognition and Brain Sciences Unit, Cambridge, United Kingdom

**Keywords:** Arcuate fasciculus, Diffusion Tensor Imaging, Language, Stroke

## Abstract

Language reorganisation following stroke has been studied widely. However, while studies of brain activation and grey matter examined both hemispheres, studies of white matter changes have mostly focused on the left hemisphere. Here we examined the relationship between bilateral hemispheric white matter and aphasia symptoms. 15 chronic stroke patients with aphasia and 18 healthy adults were studied using Diffusion Weighted Imaging data. By applying histogram analysis, Tract-Based Spatial Statistics, tractography and lesion-tract overlap methods, it was found that damage to the left hemisphere in general, and to the arcuate fasciculus in particular, correlated with impairments on word repetition, object naming, sentence comprehension and homophone and rhyme judgement. However, no such relationship was found in the right hemisphere. It is suggested that while some language function in aphasia can be explained by damage to the left arcuate fasciculus, it cannot be explained by looking at the contra-lesional tract.

## Highlights

• We investigated the role of the arcuate fasciculus (AF) in post-stroke aphasia.
• We found that left hemispheric AF damage correlates with aphasia symptoms.
• We found no evidence for a role of the right hemispheric AF in aphasia symptoms.

## 1 Introduction

Plastic reorganisation of the language system in the adult brain can occur in response to central injury at different levels. There is an on-going debate whether recovery-related language reorganisation happens only in perilesional areas (Furlan et al., 1996, Iglesias et al., 1996, Price et al., 1999, Warburton et al., 1999) or in contra-lesional areas as well (Calvert et al., 2000, Cappa et al., 1997, Weiller et al., 1995, Winhuisen et al., 2005). Some suggest that contra-lesional activation is a maladaptive reaction (Naeser et al., 2004, Price and Crinion, 2005) and can even be a predictor of poor recovery (Kurland et al., 2004). This activation might be a result of loss of inhibition to the contra-lateral hemisphere, also known as transcallosal disinhibition (Price & Crinion, 2005). Some studies demonstrated that recovery is characterised by an initial stage in which recovery-related activation can be seen in contra-lesional areas (Fernandez et al., 2004, Saur et al., 2006, Xu et al., 2004), followed by recovery-related activation in perilesional areas (Fernandez et al., 2004, Saur et al., 2006). Recovery depends on many factors such as the type of aphasia, initial severity, age, therapy given, lesion location and size (Lazar & Antoniello, 2008). More recent studies suggest that beneficial right hemispheric activation is more likely to occur in large legions (Crosson et al., 2007, Hamilton et al., 2011), when the lesion affects crucial areas for language processing (Crosson et al., 2007, Hamilton et al., 2011), and when the initial aphasia symptoms are severe (Crosson et al., 2007). Specifically, Hamilton et al. (2011) and Anglade, Thiel, and Ansaldo (2014) suggest a hierarchical model in which small lesions result in peri-lesional plasticity, larger lesions require intra-hemispheric plasticity to achieve recovery of function, and very large lesions result in right hemispheric activity, but this cannot fully compensate for loss of function in the left hemisphere (Hamilton et al., 2011). This model is supported by findings from a recent study of therapy-induced changes in brain activation following stroke (Abel, Weiller, Huber, Willmes, & Specht, 2015). Abel et al. (2015) have noted that right hemispheric (RH) compensation is more likely to occur in areas homologous to the left hemispheric (LH) lesion, and in those right hemispheric regions which show language related activation in the healthy brain (Abel et al., 2015). Lastly, Anglade et al. (2014) also suggested that language lateralisation before injury might be a major determinant for both language recovery and observed levels of RH activation, but that currently it cannot be assessed.

Previous studies looked mainly at grey matter (GM) integrity and activation. However, aphasia symptoms and their severity cannot be fully explained by GM damage, as seen on standard T1 volumetric MRI scans (Ciccarelli et al., 2008, Kim et al., 2011, Mao et al., 2007). Studies suggest that local damage can cause local fibre displacement and reorganisation of white matter (WM) as well (Heller et al., 2005, Powell et al., 2007), and that WM integrity can also influence rehabilitation potential (Meinzer et al., 2010, Rosso et al., 2014). Moreover, following a study showing the importance of symmetry across right and left hemispheric WM for language performance among healthy adults (Catani et al., 2007), Catani and Mesulam (2008) suggested that greater premorbid hemispheric symmetry may lead to better aphasia recovery, with right hemispheric tracts compensating for loss of LH function. Together, these studies highlight the importance of studying the relationship between WM damage and integrity, and aphasia symptoms. Studying WM using Diffusion Weighted Imaging (DWI) has some advantages over more traditional MRI modalities. Firstly, WM integrity (Floel, de Vries, Scholz, Breitenstein, & Johansen-Berg, 2009) and laterality (Catani et al., 2007) have already been linked to language processing. Secondly, DWI can document small changes in WM following training (Scholz, Klein, Behrens, & Johansen-Berg, 2009) and aphasia therapy (Meinzer et al., 2010, Schlaug et al., 2009, Zipse et al., 2012). However, there are only few DWI studies of post-stroke aphasia in adults. These include some interesting case-studies (Kim et al., 2011, Kwon and Jang, 2011, Selnes et al., 2002), and a few studies examining the relationship between damage to the Arcuate Fasciculus (AF) and repetition deficit (Breier et al., 2008, Kwon and Jang, 2011, Selnes et al., 2002, Song et al., 2011, Yamada et al., 2007, Zhang et al., 2010), the defining symptom of conduction aphasia (Benson & Ardila, 1996). A recent study found various measurements of speech production and naming ability to be predicted by lesion load of the AF, but not by lesion load of the uncinate fasciculus or the extreme capsule (Marchina et al., 2011).

Only few studies so far have looked at WM integrity and its relationship to aphasia symptoms in a relatively large group of aphasic stroke patients. Hosomi et al. (2009) found that while healthy adults and patients with no aphasia showed leftwards asymmetry in number of AF fibres, patients with aphasia had no such asymmetry. However, patients’ status (having aphasia or not) was defined using the relatively coarse measurements of the National Institutes of Health Stroke Scale, and imaging was done at the acute stage, when changes in both the infarct and the contra-lesional hemisphere can still occur. While acute imaging is helpful for prognosis, it is less relevant for answering questions regarding reorganisation and recovery. Another study (Forkel et al., 2014) examined changes in right and left AF of patients with aphasia, in the acute and chronic stages. The authors found that the long segment of the right, but not left, AF predicted chronic behavioural scores.

In the present study MRI images were acquired at the chronic stage, and a comprehensive language profile was defined at the same time. This allowed us to study a heterogeneous group of patients with post-stroke aphasia, by examining the relationship between language deficit and WM integrity. We examined five language tasks which cover various domains of language processing: auditory sentence comprehension, overt object naming, overt word repetition, and rhyme and homophone judgements using inner speech. Results from our previous study suggested that damage to frontal and parietal WM can affect performance of those inner speech tasks (Geva, Jones, et al., 2011).

We first examined which specific language abilities correlate with LH damage. We then examined the relationship between those specific language abilities and RH WM integrity. We hypothesised that damage to the left AF will predict language performance. In line with Catani and Mesulam (2008) we further hypothesised that WM integrity in the RH would correlate with performance. This can be due to: (1) post-stroke reorganisation, or, (2) pre-stroke individual differences which serve as a protective factor. We attempt to disentangle the two explanations by comparing RH AF parameters in patients, to a group of age- and sex-matched controls. If the controls and patients do not show differences in the RH, then it is likely that the result in patients does not represent a post-stroke reorganisation. If, however, patients have different RH AF parameters to controls, then this would suggest post-stroke reorganisation.

## 2 Materials and methods

### 2.1 Participants

15 chronic patients with aphasia (10M/5F; age range: 42–81, mean age: 64 ± 11; mean time since last stroke: 25 ± 20 months) and 18 healthy adults (12M/6F; age range: 53–75, mean age: 64 ± 6) participated in the study. All participants were native English speakers. The two groups were matched on sex and age (independent sample *t*-test for age, *p* = 0.98).

**Fig. 1:**
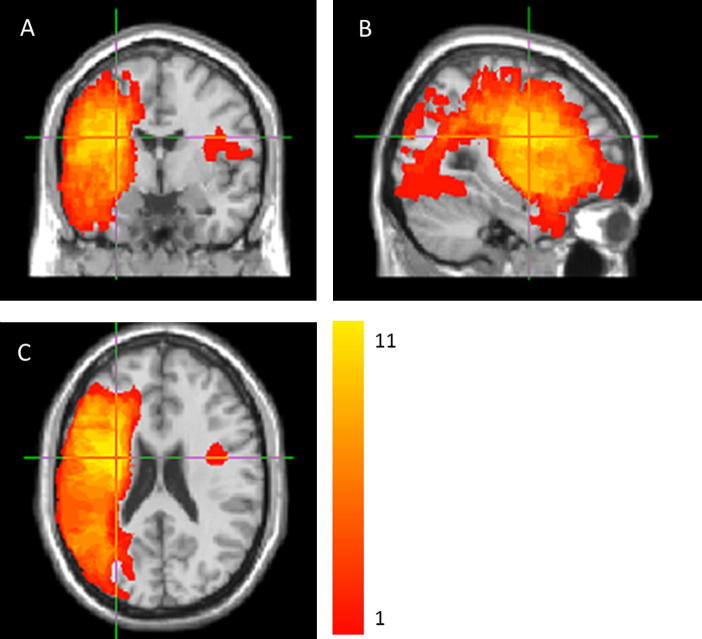
An overlay of all patients’ lesions in coronal (A), sagittal (B) and axial (C) planes. Colours represent number of patients with a lesion to a specific voxel. Warmer areas indicate areas of greater lesion overlap. Colour range runs from 1 (the lowest value in the image) to 11 (the highest value in the image). MNI coordinates of the centroid of the cluster with the largest lesion overlap: *x* = −29, *y* = −2 and *z* = 23.

All patients developed aphasia following a left Middle Cerebral Artery (MCA) territory stroke, but had no history of other neurological or psychiatric disorders. Patients with previous clinically documented right hemispheric stroke/Transient Ischaemic Attack (TIA) were excluded. The diagnosis of aphasia was based on the convergence of clinical consensus (diagnosis by a Neurologist and a Speech and Language Therapist) and the results of the Comprehensive Aphasia Test (CAT; Swinburn, Porter, & Howard, 2004), an aphasia test battery standardised for British population. All patients were tested in the chronic phase (at least 6 months following stroke). Table 1 presents demographic and clinical information for patients who completed the study. Fig. 1 presents a lesion overlap map. The study was approved by the Cambridge Research Ethics Committee and all participants read an aphasia-friendly information sheet and gave written informed consent according to the Declaration of Helsinki before participating in the study.

### 2.2 Behavioural testing

Patients were tested on the Comprehensive Aphasia Test (Swinburn et al., 2004) and the Apraxia Battery for Adults (Dabul, 1979). We analysed five tasks; three were taken from the CAT and two were adapted from the Psycholinguistic Assessments of Language Processing in Aphasia (PALPA; Kay, Coltheart, & Lesser, 1992). Participants performed all tasks without a time limit.

Patients also performed cognitive tests, including the Brixton Test of executive functions (Burgess & Shallice, 1997), the Raven Matrices (for measuring non-verbal IQ; Raven, 1938), the Rey–Osterrieth Complex Figure Test (testing visual short term memory; Meyers & Meyers, 1995) and parts of the Addenbrooke’s Cognitive Examination-Revised, testing visual–spatial abilities (Mathuranath, Nestor, Berrios, Rakowicz, & Hodges, 2000). The cognitive tests were used to exclude patients who, due to cognitive or sensory impairments, could not perform the language tasks reliably, and to allow non-linguistic modifications of task administration procedure where needed. No patients were excluded based on this set of cognitive tests.

The following tasks from the Comprehensive Aphasia Test were analysed: (1) Auditory sentence comprehension: participants were read a sentence and asked to point to one of four pictures which best fitted the sentence. The task had 16 trials. (2) Word repetition: participants were asked to repeat words read out by the examiner. This task included 16 short words. (3) Object naming: participants were asked to name 24 pictures of objects. In all three tasks, a correct answer was given two points. A delayed answer (given after more than 5 s) or a correct answer following self-correction was given one point. In the auditory sentence comprehension and the word repetition tasks, if the participant asked the examiner to repeat the question, and this was followed by a correct answer, one point was given as well.

The following tasks were adapted from PALPA: (1) Rhyme judgement: participants were asked to determine whether two written words rhymed (e.g. head-bed) or not (e.g. food-blood). The test had 60 pairs altogether. Half of the rhyming pairs and half of the non-rhyming pairs had orthographically similar endings (e.g. town–gown), whereas the other half had orthographically dissimilar endings (e.g. chair–bear), ensuring the tests could not be successfully solved based on orthography alone. (2) Homophone judgement: participants were asked to determine whether two written words sounded the same (e.g. might-mite) or not (e.g. ear-oar). This test had 40 word pairs. Items in the rhyme and homophone judgement tasks were divided into two lists. Patients performed the task on one list using inner speech and other list, using overt speech. In both cases, the patient first read both words in the pair and then gave his/her judgement for the pair. One point was given for correct answer and zero for an incorrect answer. We present here the data for the inner speech tasks only (further details on the behavioural performance of these tasks can be found in Geva, Bennett, Warburton, & Patterson, 2011).

### 2.3 Imaging data acquisition

Data were acquired using a 3T Siemens Trio scanner. Whole brain DWI data was acquired (Repetition Time (TR) = 8300 ms, Echo Time (TE) = 98 ms, field of view: 19.2 cm, axial plane, slice thickness: 2 mm, 63 slices, acquisition matrix size: 96 × 96, voxel size: 2 × 2 × 2 mm^3^) using a twice refocused spin echo sequence to reduce eddy currents (Reese, Heid, Weisskoff, & Wedeen, 2003). Diffusion sensitising gradients were applied along 12 gradient directions repeated for each of 5 *b*-values equally spaced between 0 and 1500 mm^2^/s. Other acquired sequences included a Magnetization-Prepared Rapid Acquisition Gradient Echo scan (TR = 2.3 s, TE = 2.98 ms, field of view: 240 × 256 mm, sagittal plane, slice thickness: 1 mm, 176 slices), proton density and T2-weighted scans (TR = 4.6 s, TE = 12 ms for proton density; 104 ms for T2, field of view: 168 × 224 mm, sagittal plane, slice thickness: 5 mm, 27 slices, acquisition matrix size: 240 × 320), and an axial Fluid-Attenuated Inversion Recovery scan (TR = 7.84 s, TE = 95 ms, field of view: 256 × 320 mm, axial plane, slice thickness: 4 mm, 27 slices).

### 2.4 Imaging data pre-processing

For Tract Based Spatial Statistics (TBSS) and histogram analyses, the DWI data was pre-processed with FMRIB Software Library (FSL version 5.0.4, www.fmrib.ox.ac.uk/fsl). Firstly, the skull and noise were removed from the images using FSL’s BET. Correction for eddy currents and subject motion was applied using the ‘eddy_correct’ function. Finally, the diffusion tensor model was linearly fitted to the DWI images using ‘dtifit’, and Fractional Anisotropy (FA) images were generated for each subject. Radial and Axial diffusivity (RD and AD, respectively) maps were not investigated in this study. RD and AD have been differentially linked to demyelination and axonal injury (see for example Song et al., 2002), however, reliable estimation of RD and AD is highly dependent on the ability to identify the main direction of diffusion, and therefore these metrics cannot be meaningfully interpreted in brain areas where crossing fibre populations are known to be present (e.g. Neto Henriques, Correia, Nunes, & Ferreira, 2015). Due to the nature of the injury in stroke patients, it is not reasonable to expect the white matter changes to be confined to brain regions where the assumption of a single white matter fibre population is valid and therefore interpreting any AD and RD changes would be problematic. We therefore chose to investigate FA and tract volume as quantitative metrics for the bilateral changes in white matter properties post-stroke.

### 2.5 Histogram analysis

Histogram analysis was used to compare the mean FA in each hemisphere between patients and controls, as well as evaluate the relationship between FA and the relevant behaviour measures (Mori et al., 2008). The histogram approach was chosen because it does not require the use of image registration techniques, which were not an option given the extent of the damage on the LH of some of the patients. In order to obtain RH and LH Regions of Interest (ROIs) for both patients and controls, a plane going through the midline of each subject was drawn in native space, and used to separate the right and left hemispheres into two different binary images.

The CSF was removed from the FA images using brain masks obtained from tissue class segmentation using FSL’s FAST. An in-house MATLAB script was used to generate normalised histograms for each hemisphere of each subject (i.e., the number of counts in each bin was divided by the total number of voxels so that the sum of all values within one histogram equal unity).

In order to perform statistical analysis on the histogram data, standard histogram parameters (i.e., summary measures) were extracted: mean value, which corresponds to the average FA across the whole hemisphere; peak height, which corresponds to the normalised frequency associated with the most populated histogram bin; and peak location, which was calculated as the weighted average of the top 40% values around the modal FA value.

The histogram parameters were compared between patients and controls using a two-sample *t*-test for each hemisphere. Next, correlation between each histogram parameter and the behavioural measures for patients was assessed using a General Linear Model, including all behavioural measures, as well as age, sex and lesion volume as covariates. Backward stepwise elimination was used to select the covariates which explain most of the variance seen in the histogram parameters.

The LH histogram analysis was also repeated by masking the brain area affected by the stroke in the LH of each patient, so that the histogram parameters represent only the distribution of FA values outside the lesion.

Statistical analysis was performed using SPSS version 22 (http://www-01.ibm.com/software/uk/analytics/spss/).

### 2.6 Tract Based Spatial Statistics (TBSS)

Histogram analysis can be informative when a particular pathology is expected to result in diffuse WM changes across the brain. However, it has limited value for the study of specific WM damage, as it conveys no information about the spatial localisation of the changes. For this reason, we used TBSS to further investigate FA differences in the RH. TBSS is more powerful and informative than histogram analysis since it has the ability to identify the spatial location of the WM changes. A previous study on excitatory rTMS treatment of post stroke aphasia (Allendorfer, Storrs, & Szaflarski, 2012) successfully applied standard whole-brain TBSS analysis to compare patients and controls, with only relatively small breaks in the WM skeleton on the LH. However, this approach was not feasible for our data due to the very large LH lesions in some of the patients (see Fig. 1). When whole brain registration was applied to our patients, this resulted in severely distorted data on both hemispheres. In addition, due to the extensive WM damage on the LH of patients, most of the LH white-matter skeleton was not traceable, and therefore we decided to focus on voxel wise analysis of the RH only. The RH masks generated for the histogram analysis were used again to produce RH FA maps for each subject. These RH images for all patients and controls were analysed using a standard TBSS pipeline (Smith, Jenkinson, & Johansen-Berg, 2006). As per standard procedure in TBSS, the template for image registration was chosen by aligning every FA image to every other one in order to identify the “most representative” subject. After this image registration step, each FA image was taken in turn and the average amount of warping that was necessary to align all other images to it was estimated. The “most representative” subject was chosen as the one with the smallest amount of average warping when used as a target. The mean FA skeleton, a representation of the centre of the WM tracts common to all subjects, was created for the RH only, and thresholded at FA > 0.25.

A design matrix was generated using the GLM tool in FSL. The RH images were categorised as patients or controls, and age and sex were included as nuisance covariates. Scores for the relevant behavioural measures (auditory sentence comprehension, word repetition, object naming, rhyme judgement and homophone judgement) were included as covariates of interest to investigate whether WM microstructure correlates with task performance.

Statistical analysis was performed using FSL’s tool ‘randomise’ with 1000 permutations. Threshold Free Cluster Enhancement (TFCE; Smith, Nichols, Smitha, & Nicholsb, 2009) was applied for correction of multiple comparisons. The threshold for statistical significance was *p* < 0.05.

### 2.7 Tractography

To characterise the right and left AF directly, we used streamline tractography and MRtrix (J-D Tournier, Brain Research Institute, Melbourne, Australia, http://www.brain.org.au/software/; Tournier, Calamante, Gadian, & Connelly, 2004).

**Fig. 2:**
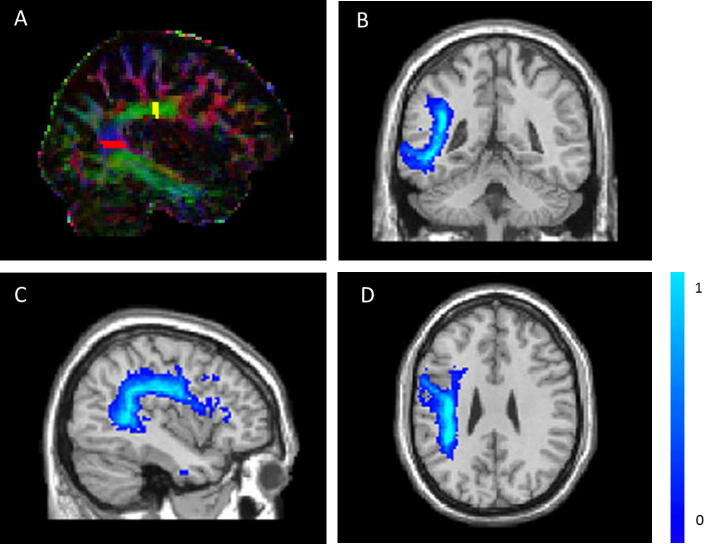
An example of a seed (yellow) and target (red) Regions of Interest used for tracking the Arcuate Fasciculus (A); and a probabilistic left hemispheric Arcuate Fasciculus map based on 17 healthy participants, seen from coronal (B), sagittal (C) and axial (D) views (colour scale represents probabilities from 0 to 1).

For each participant, seed and target ROIs were drawn on the right and left hemispheres, in native space, according to a set protocol based on Liégeois et al. (2013). The seed was placed in the dorsal portion of the AF, approximating the location of the arcuate fasciculus ‘bottleneck’ (as in Galantucci et al., 2011), posterior to the central sulcus, while avoiding being directly inferior to cortico-spinal descending tracts. It was drawn on two adjacent slices, in coronal plane, on voxels coded green on the eigenvector map – indicating an anterior–posterior direction. The target was placed on the ascending portion of the AF. It was drawn on two adjacent slices, in axial plane on voxels coded blue on the eigenvector maps, indicating dorsal–ventral direction (see Fig. 2). All seeds and targets were drawn by SG, who was blinded to the participants’ language profile. For every tract, 100,000 streamlines were generated from the seed ROI, and only those streamlines which connected with the target ROI were retained. In cases where spurious tracts were identified, exclusion ROIs were drawn in native space and streamlines were re-generated, using the seed, target and the exclusion ROIs. The following exclusion ROIs were used when necessary: (1) exclusion of descending cortico-spinal tracts using an axial ROI drawn at the level of the internal capsule; (2) exclusion of ascending cortical tracts using an axial ROI superiorly to the AF; (3) exclusion of tracts belonging to the Inferior longitudinal fasciculus using a coronal or sagittal ROI drawn on the occipital lobe; and, (4) exclusion of tracts crossing between the hemispheres using a sagittal ROI drawn on the midline.

The number of tracts which successfully reached the target ROI was divided by the number of tracts generated to define the percentage of success. The resulting tracts were transformed into Tract Density Images (TDI), where each voxel value represents the proportion of streamlines that pass through that voxel relative to the overall number generated. These TDI images where subjected to thresholding to reduce the contribution of spurious streamlines, and then converted to binary masks. The threshold was set to 0.001, i.e., at least 100 streamlines contained within any given voxel. The tract volume corrected for overall Intracranial Volume (ICV) and mean FA were determined within the binary mask in each subject and hemisphere.

Group comparisons were done using independent and paired sample *t*-tests, and correlations using Pearson’s *R*.

### 2.8 Lesion-Tract Overlap (LTO)

To further characterise the left hemispheric WM damage, we adopted a method which evaluates the integrity of the LH AF specifically. Following Marchina et al. (2011), Lesion-Tract Overlap (LTO), a combined variable of lesion site and size, was defined for our patient group. Lesions were defined manually as described in Geva, Baron, Jones, Price, and Warburton (2012). In short, lesions were defined using the ROI facility in Analyze 7.5 software (Mayo Biomedical Imaging Resource, Mayo Clinic, MN). One author (SG) traced the lesions manually on patients’ individual T2-weighted scans, in native space, while consulting other sequences. Lesions were identified on a slice by slice basis. The drawn lesions were validated by a trained neurologist (EAW) who was blinded to the patients’ diagnoses. If atrophy was identified in the RH (despite including only patients who did not have a documented right hemispheric stroke/TIA), this right hemispheric atrophy was included in the lesion definition. Binary masks were made from the lesions (Brett, Leff, Rorden, & Ashburner, 2001) using MRIcron 2009 (Rorden, Karnath, & Bonilha, 2007), and were used to calculate lesion volume. Lesion volume was corrected for overall ICV. The binary lesions were then used as masks, and each patient’s T1-weighted image was normalised into MNI space using SPM5 (Wellcome Trust Centre for Neuroimaging, London, UK, www.fil.ion.ucl.ac.uk/spm). The normalisation parameters were then applied to the binary lesion. The reconstruction of the LH AF was successful for 17 controls (see Table 2). The thresholded binary AF masks for these 17 controls were normalised into MNI space using SPM5, and summed to generate a fibre map using MATLAB (see Fig. 2). Voxel intensities ranged between 0 (when the voxel is not part of the AF for any control) and 17 (when the voxel is part of the AF for all controls). This image was then divided by 17, so that each voxel now represented the probability of it being part of the AF. For each patient, the LTO was calculated by overlapping the normalised binary lesion on the probabilistic AF map and summing the intensities of all the intersecting voxels:

$$\text{LTO}=\overset{n_{\max}}{\underset{n=1}{\sum }}\frac{1}{17}I_{n}V_{\mathrm{voxel}}$$

where *n_max_* is the total number of intersecting voxels, and *I_n_* is the intensity of the *n*^th^ voxel in the probabilistic AF map. *V_voxel_* represents the volume of each voxel, and therefore LTO is expressed in mm^3^.

## 3 Results

### 3.1 Behavioural performance

All behavioural results are presented in Table 3. All patients completed the three CAT tasks. Possible range and cut-off score for defining impaired function are presented in Table 3. The mean score on the auditory sentence comprehension was 24 ± 5.78 (range: 15–32). The mean score on the word repetition task was 24.33 ± 10.44 (range: 0–32). The mean score on the object naming task was 30.60 ± 16.17 (range: 0–48).

One patient did not complete the homophone judgement task and three did not complete the rhyme judgement task (Table 3). The mean score on the homophone judgement task was 73 ± 22% correct, and on the rhyme judgement 77 ± 17% correct.

Age did not significantly correlate with any of the behavioural scores (Pearson correlation, *p* > 0.3 for all), and there was no significant difference between males and females on any of the behavioural tests (*t*-test, *p* > 0.3 for all).

Right hemispheric atrophy was found for two patients (P08 and P12, see Table 1). These patients were not previously diagnosed clinically as having right hemispheric stroke/TIA and therefore did not meet our exclusion criteria. However, in order to evaluate their contribution to the various results, we performed analyses with and without these two patients, when relevant.

### 3.2 Histogram analysis

The comparison between patients and controls revealed no significant difference on the RH for mean FA (*p* = 0.228), peak height (*p* = 0.070) and peak location (*p* = 0.064), while the LH comparison revealed a highly significant difference for all three histogram parameters with *p* < 0.001. The RH analysis was repeated without the two patients which were found to have RH atrophy, and all comparisons remained non-significant (mean FA *p* = 0.347, peak height *p* = 0.132, peak location *p* = 0.101).

Within the patient group, LH peak height and peak location did not correlate significantly with any of the behavioural measures. Mean FA was found to correlate positively with auditory sentence comprehension (*p* = 0.001, *R*^2^ = 0.584), object naming (*p* = 0.013, *R*^2^ = 0.416), rhyme judgement (*p* = 0.0184, *R*^2^ = 0.442) and homophone judgement (*p* = 0.008, *R*^2^ = 0.461), but not word repetition (*p* = 0.314, *R*^2^ = 0.084). Mean FA also correlated negatively with lesion size (*p* < 0.001, *R*^2^ = 0.655). However, when model selection was applied, the best model to explain the variability in mean FA included only age and lesion size (*p* < 0.001 for lesion size and *p* = 0.024 for age).

This analysis was repeated using the lesions as masks, such that the histogram parameters reflected the FA distribution outside the affected brain region for each subject. Larger lesions are likely to cover more WM, which would result in lower mean FAs outside the lesion independently of whether or not there is WM damage beyond the visible lesion. For this reason, we included lesion size in the general linear model for this analysis, as well as all the behavioural metrics, age and sex. Backward stepwise elimination found that the model which explained most of the variance in mean FA outside the lesion was the one with lesion size as the only factor (*p* = 0.006 for effect of lesion size).

The same analysis was repeated for the three histogram metrics on the RH of patients and no significant correlations with behaviour were found (*p* > 0.1 for all).

### 3.3 Tract Based Spatial Statistics (TBSS)

For the TBSS analysis of the RH we excluded the two patients who were found to have damage in the RH despite not being previously diagnosed as having suffered from RH stroke/TIA, and no significant differences between patients and controls were present (*p* > 0.05, TFCE corrected). Also, no significant correlations were found between RH FA and the behavioural measures for the patients.

### 3.4 Tractography

**Fig. 3:**
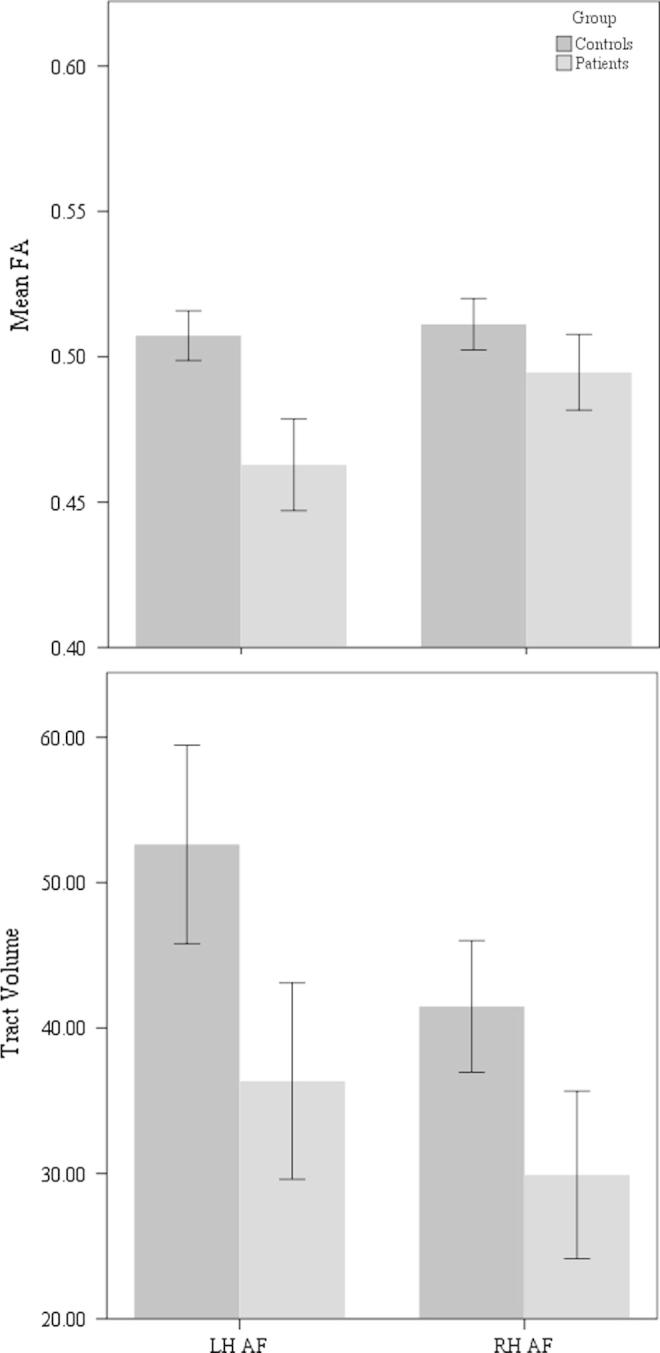
Mean Fractional Anisotropy (FA) values and tract volume (corrected for intracranial volume) of the right and left hemispheric Arcuate Fasciculus, in patients and controls. Bars represent ±1 standard error.

Tractography was attempted in both hemispheres and success rates (Table 2) are similar to previous publications (Brandstack et al., 2013, Catani et al., 2007, Lebel and Beaulieu, 2009). In the LH, patients had lower FA (*p* = 0.008, *t* = 2.9) when compared to controls, but there was no difference in tract volume (*p* = 0.31, *t* = 1.0). There were no significant differences between patients and controls’ FA values or tract volume in the RH (*p* = 0.32, *t* = 1.02; *p* = 0.16, *t* = 1.44; respectively). See Fig. 3.

Patients without tractable LH AF did not differ in age (*p* = 0.837, *t* = 0.2) and time since stroke (*p* = 0.151, *t* = 1.5) from those patients for whom the LH AF was tracked. However, they had larger lesions (*p* = 0.022, *t* = 2.6). It was found that patients without tractable LH AF performed significantly worse than patients with a tractable LH AF, on auditory sentence comprehension (*p* = 0.006, *t* = 3.3), object naming (*p* = 0.001, *t* = 4.6), word repetition (*p* = 0.023, *t* = 2.7), homophone judgement (*p* = 0.014, *t* = 2.8), and rhyme judgement (*p* = 0.014, *t* = 2.9). To check whether the difference in behavioural performance can be explained by the difference in lesion volume we added lesion volume as a covariate in the analyses. It was found that the differences between patients with and without LH AF remained significant.

Looking at the RH AF, it was found that patients for whom the LH AF was tracked did not differ in RH AF volume or mean FA, from those patients for whom the LH AF was not tracked (*p* = 0.654, *t* = 0.5; *p* = 0.996, *t* = 0, respectively).

All results remained similar when excluding the two patients for whom right hemispheric atrophy was detected, although in some cases *p* values were higher.

In summary, the patients as a group did not differ from their matched controls on measurements of RH AF. Moreover, patients for whom it was impossible to successfully track the LH AF performed worse on all behavioural measurements, but did not differ from other patients in their RH AF measurements.

### 3.5 Lesion-Tract Overlap

This analysis allowed us to evaluate the degree of damage to the LH AF in all patients, including those for whom we could not track the LH AF using tractography. LTO values are shown in Table 1.

**Fig. 4:**
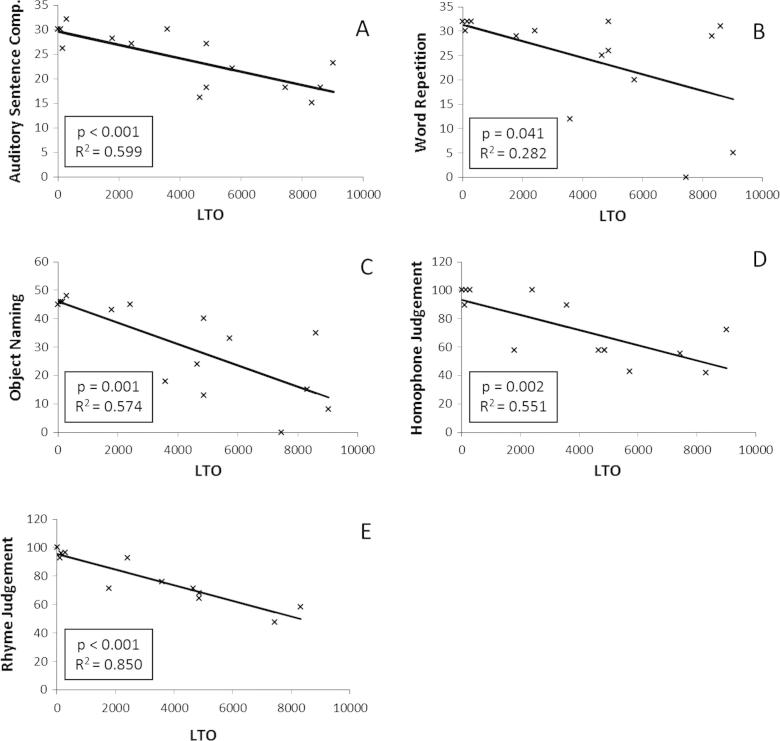
Correlations between Lesion-Tract Overlap (LTO) and behaviour. *Y* axis represents raw scores (A–C) or percentage correct (D, E).

A regression analysis was performed, using LTO and lesion size as predictors for auditory sentence comprehension, word repetition, object naming, rhyme and homophone judgement. Both LTO (*p* < 0.001, *R*^2^ = 0.599) and lesion size (*p* < 0.001, *R*^2^ = 0.625) were found to be good predictors of auditory sentence comprehension. LTO was found to significantly predict word repetition scores (*p* = 0.041, *R*^2^ = 0.282), whereas lesion size was found to be non-significant (*p* = 0.052, *R*^2^ = 0.259). LTO significantly predicted object naming scores (*p* = 0.001, *R*^2^ = 0.574) as did lesion size (*p* = 0.003, *R*^2^ = 0.497). LTO and lesion size were also found to significantly predict rhyme judgement (*p* = 1.98 × 10^−5^, *R*^2^ = 0.85 and *p* = 5.65 × 10^−4^, *R*^2^ = 0.711, respectively) and homophone judgement scores (*p* = 0.002, *R*^2^ = 0.551, and *p* = 0.004, *R*^2^ = 0.515, respectively). See Fig. 4.

Lastly, we examined whether LH damage can predict RH AF integrity, and whether RH AF integrity can predict behaviour. Regression analysis showed that LTO was not a significant predictor for mean FA or volume of the RH AF (*p* = 0.882, *R*^2^ = 0.005; *p* = 0.162, *R*^2^ = 0.173, respectively). Secondly, regression analyses were performed, using volume and mean FA of the RH AF as predictors of the behavioural measures. Again, all models were found to be non-significant (*p* > 0.05 for all).

## 4 Discussion

In this study we attempted to understand the influence of WM damage in the LH, and WM integrity in the RH, on aphasia symptoms. Firstly, using three different methods, we investigated the relationship between left hemispheric WM damage and language function. Using histogram analysis, which can evaluate the damage in all patients and across the entire hemisphere, we have found that overall WM damage, as reflected in mean FA, correlated with behavioural measurements for all tests other than word repetition. It should be noted that in our population word repetition was relatively intact and indeed, none of our patients was diagnosed clinically as having aphasia profile resembling conduction aphasia. However, lesion size had an overwhelming effect and when model selection was applied it was found that age and lesion size could best explain the variability in our data. This replicates many previous studies showing that lesion size is related to behavioural impairment (Lazar and Antoniello, 2008, Szaflarski et al., 2013).

Next, we used tractography to look at the integrity of the AF itself. This method allows us to segment the AF for each subject, and therefore compare measurements taken directly from WM tracts which comprise the AF. However, in many stroke patients with left MCA territory damage, the left AF cannot be completely tracked due to lesion location. We found that patients for whom the left AF could be tracked performed significantly better than those for whom the AF could not be tracked. Importantly, this difference was significant over and above the influence of lesion size.

Lastly, we examined the influence of degree of damage to the left hemispheric AF on aphasia symptoms using a lesion load technique. Once again we found that the degree of damage to the left AF predicted behavioural performance for most measurements. Thus, using techniques that measure the damage to the AF, rather than looking at the entire LH, we replicated the results obtained using the histogram analysis.

These results are in agreement with Rosso et al. (2014) who showed that the degree of behavioural improvement on a naming task, following right IFG cathodal tDCS, correlated with left arcuate fasciculus FA. Interestingly, in Rosso et al. (2014) this was the case only for patients with damage in left Broca’s area.

In summary, we used three different techniques, each with its own advantages and disadvantages, and replicated the findings suggesting that WM damage to the left hemispheric AF is correlated with various measurements of language function. Our tractography analysis suggests that this correlation is significant over and above the influence of lesion size. The role of the left AF in language processing is relatively uncontroversial (Dick & Tremblay, 2012). It has been suggested that it comprises the dorsal languages stream (Hickok and Poeppel, 2007, Poeppel and Hickok, 2004), and its role in repetition (Breier et al., 2008, Fridriksson et al., 2010, Kümmerer et al., 2013), naming (Marchina et al., 2011), sentence comprehension (Brauer et al., 2011, Friederici, 2009) and homophone and rhyme judgement (Geva, Jones, et al., 2011) has been suggested before. Our results therefore add to the cumulated data showing involvement of the left AF in tasks requiring integration between auditory and motor speech processing (Kümmerer et al., 2013, Parker Jones et al., 2014, Saur et al., 2006).

We then turned to examine the relationship between language function and right hemispheric WM integrity in our patient cohort. We used histogram and whole-hemisphere TBSS analyses, and found no correlation between right hemispheric histogram/FA values and language function, and no differences between patients and a group of healthy age-matched controls. This latter finding suggests that our patients, as a group, did not have diffuse damage (which can be found, for example, in cases with vascular dementia or multiple strokes) or right hemispheric Wallerian Degeneration.

Since we have demonstrated a correlation between left hemispheric AF damage and performance on various language tests, we next focused on the right hemispheric AF using tractography. However, in contrast to the left hemispheric findings, we found no evidence for right hemispheric involvement in language function. This finding is supported by results from two types of analyses. The first, looking at stroke patients alone, found no significant correlation between behaviour and right hemispheric AF measurements, no difference in right hemispheric AF measurements between those patients with less severe left hemispheric damage and those with more severe damage (as seen by the ability or inability to track the left hemispheric AF), and no relationship between degree of left AF damage (measured using LTO) and right hemispheric AF measurements. The latter results might seem to contradict the models by Hamilton et al. (2011) and Anglade et al. (2014) which suggest lesion size to be an important determinant for right hemispheric involvement. However, lesion site is also a major contributor in these models, and might even dominate the results. These results are in agreement with findings by Szaflarski et al. (2013), who showed that behaviourally recovered patients showed greater activation in left, but not right, cerebral areas, during a functional MRI (fMRI) semantic decision task, and that right hemispheric activation was associated with poorer recovery. The second line of analysis compared the stroke patients with their matched control group. Here we found no differences in right hemispheric AF measurements between groups. Thus, using various techniques and analyses we found no evidence for right hemispheric involvement in language function in our chronic stroke patients. These results are in contrast with Forkel et al. (2014) who found RH WM parameters to be predictive of chronic symptoms. However, it should be noted that Forkel et al. did not directly compare the predictive value of the right and left hemispheres, therefore it cannot be determined that the RH WM integrity is indeed significantly more predictive than LH one. In addition, in their study, the AF could be tracked for all 16 left MCA territory stroke patients. This is somewhat unusual considering the anatomy of the MCA, suggesting that this cohort has relatively confined lesions. On the other hand, Forkel et al. used a longitudinal design which is better suited to capture plastic changes which occur over time, especially in highly variable anatomical structures such as the AF (for example Brandstack et al., 2013). Studies on larger patient cohorts should be performed in order to shed light on the role of the left and right WM tracts in aphasia recovery in various types of patients.

The involvement of the RH in language processing has been discussed widely in recent years. Many imaging studies show brain activation in the RH during various language tasks, ranging from lexical decision (Newman, 2012), overt picture naming (Diaz et al., 2014) and sentence comprehension (De Vega et al., 2014), to overt picture description in a communicative context (Schoot, Menenti, Hagoort, & Segaert, 2014), to name only a few tasks. However, it is important to evaluate how these occurrences of right hemispheric activation are interpreted. Recent reviews point to two types of processes in which the RH is involved in language processing. The first is low level processing. For example, Price (2012) suggests that low level auditory processing occurs in bilateral planum temporale and right superior temporal gyrus. The second type of processes is those which are meta-cognitive and not language-specific. For example, in her reviews of imaging studies, Price, 2010, Price, 2012 suggests that right inferior frontal activation in language studies represents conflict resolution or inhibition of irrelevant response. Lastly, notice that recent models of speech processing (Friederici, 2011, Hickok and Poeppel, 2007) suggest that only the ventral language stream is bilateral, while the dorsal stream, which relies largely on the AF, is strongly left lateralised.

Hence, it seems that the involvement of the RH in language processing is often restricted to processes which are not core linguistic processes, a finding which might explain the lack of right hemispheric involvement found in our study. Since in post-stroke aphasia core linguistic processes are impaired, right hemispheric support might only have limited contribution to recovery of function.

This study suffered from the general limitations of diffusion tensor modelling and tractography approaches (for a recent review with specific reference to the domain of language see Campbell & Pike, 2013). These included the inherent assumption of a single fibre population within each voxel, and consequent inability to resolve fibre crossings. Because only 12 non-colinear gradient directions were acquired, we were not able to perform more sophisticated modelling which would allow us to overcome these limitations, and instead we applied thresholding and exclusion masks to ensure optimised reconstruction of the AF for each subject. In theory, only 6 non-colinear directions are required to estimate the main direction of diffusion, and several studies have previously used 16 directions or less to accurately perform tractography analyses in clinical populations (Kunimatsu et al., 2003, Nelles et al., 2008, Pagani et al., 2005, Yamada et al., 2004). However, the limited number of directions might be responsible for some cases in which reconstruction of the AF was not successful, though we expected it to influence the patient and control group similarly.

A second caveat is that behavioural and imaging data were acquired only at the chronic stage. Since most changes occur in the brain at the acute stage, studying chronic patients allowed us to look at a more stable system (Saur et al., 2006). In order to evaluate the state of the system before the injury we compared our group of patients to sex- and age-matched healthy adults. However, since the lateralisation of the AF varies largely between individuals (Catani et al., 2005, Catani et al., 2007, Vernooij et al., 2007) this method is relatively limited. This documented variability lead Berthier, Lambon Ralph, Pujol, and Green (2012) to suggest that pre-stroke lateralisation can influence the relationship between post-stroke AF lateralisation and language function, and might explain negative results.

Moreover, this study design did not allow us to directly examine the changes which occur following stroke at the individual level. A longitudinal study would allow assessing the relationship between WM changes and recovery more directly. It also should be noted that correlating aphasia symptoms with left AF parameters at the chronic stage does not allow us to disentangle whether it is the acute WM integrity, or its longitudinal plasticity, which best explains the level of behavioural ability. Many studies show longitudinal and therapy-induced changes in WM parameters as measured using Diffusion Tensor Imaging (DTI) (Meinzer et al., 2010, Schlaug et al., 2009, Scholz et al., 2009, Wan et al., 2014). This brings up the hypothesis that a longitudinal study might reveal right hemispheric contributions to recovery which could not be documented in this study.

Thirdly, in this study we focused on the AF due to its established role in language processing (Dick and Tremblay, 2012, Marchina et al., 2011). However, in order to fully understand aphasic symptoms and recovery, future studies should investigate other association fibres which might support language processing (Jang, 2013), such as the uncinate fasciculus, middle longitudinal fasciculus and inferior longitudinal fasciculus, which are suggested to make the ventral language processing route (Dick & Tremblay, 2012).

Lastly, due to stroke being more common among males, our cohorts (both patients and controls) was predominantly male. While this is more representative of the stroke population than a cohort with equal number of males and females, it might have also biased our results, since some segments of the AF are suggested to be more left lateralised in males (Catani et al., 2007, Thiebaut de Schotten et al., 2011). This limits our ability to generalise our results to females, and we suggest that future studies include larger number of female patients, and explore the influence of patients’ sex on outcome.

In conclusion, this study characterised WM integrity and its relationship to language impairments in a representative group of patients with post-stroke aphasia using various techniques. Each technique has its advantages and disadvantages and together they provide a fuller picture of the influence of left hemispheric WM damage on language function. We have found LH WM damage to correlate with language impairments in the domain of production, comprehension and repetition, and while interpreting the null results with cautious, we found no evidence for a link between right hemispheric WM integrity and language function. Future longitudinal studies should explore the influence of bilateral WM on language function following stroke in more detail, while attempting to obtain converging evidence using various techniques, similarly to the methods implemented here.

## Figures and Tables

**Table 1:** Demographic and clinical information for participating patients.

Patient	Age	Sex	Previous stroke^a^	Last stroke^b^	Stroke type^a^	Lesion size (mm^3^)	LH AF tracked	LH LTO	RH AF tracked
Time since stroke (months)	
P01	78	m	50 (LH; i)	20	i	124.68	No	7.33	Yes
P02	73	m	29 (not seen on MRI)	18	i	68.73	Yes	8.09	Yes
P03	81	m	72 (LH; i)	19	i	129.48	No	22.85	Yes
P04	55	m		48	i	197.17	No	46.39	Yes
P05	69	m		22	i	198.54	No	58.40	Yes
P06	62	m		10	h	9.95	Yes	3.51	Yes
P08	70	m		87	i	224.09	No	105.46	Yes
P09	78	f		9	i	0.86	Yes	0.45	Yes
P10	62	m		16	i	127.46	Yes	74.98	Yes
P12	51	f		24	i	161.10	No	113.72	Yes
P13	49	f		20	i	4.68	Yes	3.58	Yes
P14	42	f		13	i	101.81	No	83.84	Yes
P15	53	f		36	i	201.28	No	177.60	No
P16	69	m	TIAs	25	i	8.76	No	8.25	Yes
P17	66	m		11	i	49.90	No	49.90	No

AF – Arcuate Fasciculus; LH – Left hemisphere; LTO – Lesion-Tract Overlap; RH – Right hemisphere.

a For patients who had more than one stroke, time since the first stroke is indicated. In brackets: type of that stroke (i = ischaemic; h = haemorrhagic, RH = right hemisphere; LH = left hemisphere).

b First stroke for patients who had only one, second for those patients who had a previous stroke. Last stroke was left hemispheric in all cases and the cause of the language deficits.

**Table 2:** Tractography success rates.

	Patients		Controls	
LH	RH	LH	RH
Males	3/10	9/10	11/12	9/12
Females	2/5	4/5	6/6	4/6
Total	5/15	13/15	17/18	13/18

LH – Left hemisphere; RH – Right hemisphere.

**Table 3:** Patients’ behavioural scores.

	Auditory sentence comprehension	Word repetition	Object naming	Homophone judgement	Rhyme judgement
Possible range	0–32	0–32	0–48	0–100%	0–100%
Cut-off score^a^	27	29	43	N/A	N/A
P01	16	25	24	57.9	71.4
P02	28	29	43	57.9	71.4
P03	18	26	13	57.7	67.9
P04	18	31	35	N/A	N/A
P05	18	0	0	55.3	47.4
P06	32	32	48	100.0	96.6
P08	23	5	8	72.4	N/A
P09	30	32	45	100.0	100.0
P10	27	30	45	100.0	92.9
P12	22	20	33	42.9	N/A
P13	26	32	46	100.0	96.0
P14	27	32	40	57.9	64.3
P15	15	29	15	41.7	58.2
P16	30	30	46	89.5	92.9
P17	30	12	18	89.5	75.9

a Cut-off score is used for defining impaired function.
